# SARS-CoV-2 and work-related transmission: results of a prospective cohort of airport workers, 2020

**DOI:** 10.47626/1679-4435-2020-681

**Published:** 2021-03-03

**Authors:** Jeadran N. Malagón-Rojas, Marcela Mercado, Claudia P. Gómez-Rendón

**Affiliations:** 1 Doctorado en Salud Pública, Universidad El Bosque, Bogotá, Colombia; 2 Dirección de Investigación, Instituto Nacional de Salud, Bogotá, Colombia

**Keywords:** COVID-19, SARS-CoV-2, working conditions, airports, occupational health

## Abstract

**Introduction:**

The coronavirus disease 2019 (COVID-19) pandemic has spread rapidly around the globe. Even though multiple strategies are available for controlling infectious respiratory diseases, the current approach for managing this pandemic is the prevention of person-to-person transmission. Despite the quarantine strategy, some work positions must remain active, such as airport personnel.

**Objectives:**

To identify risk factors for COVID-19 transmission among workers at the El Dorado, Luis Carlos Galán Airport from March to July 2020.

**Methods:**

This is a prospective cohort study with workers of the El Dorado International Airport, in Bogotá, Colombia. A sociodemographic questionnaire was for searching for symptoms associated with COVID-19 and other risk factors. Nasopharyngeal swabs were collected for determining the presence of COVID-19. In order to identify seroconversion, we used an automated chemiluminescent immunoassay for anti-SARS-CoV-2 IgM and IgG antibodies. Patients with positive results were followed-up for 21 days.

**Results:**

We observed an incidence of infection of 7.9%; most cases were asymptomatic. The main risk factor associated with infection was the duration of daily commute (relative risk 1.02 [95% confidence interval, 1.002-1.041]).

**Conclusions:**

We observed asymptomatic infection by COVID-19 among airport workers. Future research should contribute with knowledge for developing strategies that guarantee the protection of airport workers.

## INTRODUCTION

Worldwide government actions to counteract the transmission of coronavirus disease 2019 (COVID-19) and limit the interaction between people include a series of control measures such as the closure of educational institutions, trade blocks, air and land transport restrictions, and social isolation.^1^ Even though most companies and businesses were closed or implemented remote working strategies, workers of several sectors had to continue active, mainly in health care, food sales, banking services, public services, and transportation.^2^

Most infection prevention actions have focused on health care workers, since they constitute the first line of action in a pandemic.^3,4^ However, as the pandemic progressed, workers of other sectors such as fast food, restaurants, security, and transportation were identified as being at an increased risk of exposure to infected persons due to their large number of daily contacts.^5^ Airport personnel, in particular, perform a large number of activities where person-to-person contact and attention to the public are implicit, and cannot choose to switch to remote work.^6^

In this group of workers, at least 2 components have been identified to increase the risk of infections at airports.^7^ The first is related to the great mobility of passengers from different latitudes who remain concentrated for long periods in interchange areas. The second comprises the ignorance regarding the health status of travelers and the absence of devices that assess signs suggestive of infection, potentially favoring transmission. There are documented reports of virus transmission in airports: One of them refers to a series of cases of infection by the Middle East respiratory syndrome coronavirus 2 (MERS-CoV-2) at London Heathrow Airport in 2014.^8^ In this study, among the studied contacts, 5 people reported respiratory symptoms 14 days after the flight in question. A measles outbreak occurred in the same year on a trip from the Philippines to the United Kingdom, connecting in the Netherlands. The analysis identified secondary transmission in two workers at Amsterdam Schiphol International Airport and then in passengers who shared a flight from the Netherlands to the United Kingdom.^9^ During the 2009 H1N1 pandemic, airport workers also went through transmission outbreaks. In New Zealand, a series of cases compatible with influenza were identified in a flight from Mexico City to Auckland, connecting in Los Angeles.^10^ Five cases of H1N1 infection were confirmed in airport workers.^11^

In Colombia, the El Dorado Luis Carlos Galán Sarmiento International Airport is located in the capital Bogotá and receives approximately 30 million passengers per year. Its operation is guaranteed by a team of 25 000 workers and 60 companies from different sectors. The work areas are divided into cargo, airline personnel, flight crews, immigration, cleaning, security, food providers, airport health service, and others.^12^ El Dorado International Airport is not only the most important air terminal in the country but also the third connection hub with the biggest traffic of passengers from Europe and North America.^12^ Consequently, this airport is crucial in determining the risk of transmission of diseases such as COVID-19.

Due to the COVID-19 pandemic, the airport closed its commercial operations on March 22, 2020. However, it continued with the transportation of supplies and humanitarian flights, which has demanded that a large part of its workers continue working in person despite the risk of infection. Therefore, the objective of this work was to identify the risk factors for SARS-CoV-2 infection in a sample of workers at an international airport.

## METHODS

A prospective cohort was designed in a group of workers at the El Dorado, Luis Carlos Galán International Airport in Bogotá. The study followed the recommendations of the Strengthening the Reporting of Observational Studies in Epidemiology (STROBE) statement^13^ and was performed between March 22 and June 1, 2020.

A call for the study was made through the airport’s human resources office. As inclusion criteria, we considered: i) airport workers aged between 18 and 70 years; ii) workers with a valid contract and performing on-site work at the time of the study. Exclusion criteria were defined as: i) workers with close contacts who tested positive for SARS-CoV-2 on an extra-labor basis (family members at home, extended family, and others); ii) workers who traveled abroad for non-work reasons and returned between March 1 and March 20, 2020; iii) workers who stated that they did not want to participate in the study.

### SAMPLE

Based on estimates from the Ministry of Health on the incidence of SARS-CoV-2 infections in the Colombian population, the sample size was estimated using OpenEpi^®14^ and consisted in 198 workers with a 95% confidence interval (95%CI) and 3% precision.

Sociodemographic variables, working conditions, risk perception of SARS-CoV-2 transmission, travel history, history of influenza vaccination, contacts, as well as the results of serological IgM/IgG and real time-polymerase chain reaction (RT-PCR) tests for SARS-CoV-2 were considered.

### INFORMATION SOURCES

#### Risk assessment matrix

We used a matrix for evaluating hazards and measuring the occupational exposure risks for each position and role. This matrix identified the type of exposure source, evaluating the duration and type of exposure that workers experienced (direct contact with drops or aerosols, indirect contact with contaminated surfaces). From this, the risk levels for SARS-CoV-2 contagion were stratified as high, medium, and low for each position.

#### Sociodemographic characterization survey and epidemiological report form

A questionnaire was used for assessing sociodemographic variables, the presence or absence of symptoms, use of personal protective equipment, and risk perception. The questionnaire was created based on the instruments recommended by the World Health Organization (WHO) for characterizing occupational exposure to SARS-CoV-2^15^ and was subjected to content validation by 3 experts in occupational health and safety and biosecurity.^16^

Additionally, participants filled out the epidemiological report form for acute respiratory infection with a new virus, code 346 of the Colombian National Health Institute.^17^

### BIOLOGICAL SAMPLES

Each worker included in the study had a nasopharyngeal swab sample taken to determine viral RNA by RT-PCR. The sample was taken by personnel trained according to techniques of the National Health Institute, as described in the Guide for Laboratory Surveillance of Influenza Virus and other Respiratory Viruses.^17^

Additionally, each participant had a 5-mL blood sample taken; it was centrifuged and stored for identification of the presence of serum anti-SARS-CoV-2 antibodies.

### SAMPLE PROCESSING

Processing of the collected samples was performed by the RT-PCR laboratory of the National Health Institute’s Research Department. Viral RNA detection was performed according to the Berlin protocol, standardized by the RT-PCR laboratory of the National Health Institute’s Research Directorate.^18^

Antibody identification was performed using an automated chemiluminescent immunoassay for anti‐SARS‐CoV‐2 IgM and IgG antibodies.^19^ The test was read by 2 independent observers and the results were recorded in laboratory logbooks.

### FOLLOW-UP

Workers who tested positive for SARS-CoV-2 were followed up at home by 2 researchers on days 7, 14, and 21 after the first RT-PCR test. At each visit, new nasopharyngeal swab and venous blood samples were taken. Additionally, a health status evaluation was performed by a physician.

### DATA ANALYSIS

The survey data and epidemiological records, as well as RT-PCR and serology results, were recorded in a Microsoft Excel 2019 spreadsheet. Data analyses were done using the SPSS software, version 25.0.

For quantitative variables, results were expressed as means and standard deviations. For qualitative variables, results were presented as frequencies and percentages. A bivariate analysis was performed for comparing the nominal or ordinal variables regarding the presence or absence of secondary SARS-CoV-2 infection; the analysis used a Pearson’s chi-squared test with Yate’s correction, or a Fisher’s exact test for values under 5.^20^

A Poisson regression model was used, considering that the event had a low frequency.^21,22^ Risk ratios (RR) with 95%CI were used to assess differences between groups using negative responses as a benchmark. The level of statistical significance was p < 0.05.

### ETHICAL CONSIDERATIONS

The project was approved by the Research Ethics and Methodologies Committee (CEMIN) of Colombian National Health Institute under number 012/2020. Written informed consent was provided before sampling. Results were reported to the participants, who received follow-up, alarm signs, and home recommendations to prevent transmission.

## RESULTS

### SOCIODEMOGRAPHIC CHARACTERISTICS OF THE POPULATION

The study included a total of 212 workers. Most of them were male (73.1%, n = 155), mixed-race (52.4%, n = 95), of average socioeconomic level (3) (62.4%, n = 130), and had technical education (39.2%, n = 83) (Table 1).

**Table 1:** Sociodemographic characteristics of the sample of airport workers

Characteristics	Total	Sex	
		Female	Male
	% (n)	% (n)	% (n)
Age, years (mean ± SD)	36.3 ± 8.2	33 ± 6.0	37 ± 9.0
Blood type			
A (-)	0.9 (2)	0.5 (1)	0.5 (1)
A (+)	29.7 (63)	7.1 (15)	22.6 (48)
AB (+)	0.9 (2)	0.5 (1)	0.5 (1)
B (-)	0.9 (2)	0.0 (0)	0.9 (2)
B (+)	6.1 (13)	2.8 (6)	3.3 (7)
O (-)	3.8 (8)	0.9 (2)	2.8 (6)
O (+)	57.5 (122)	15.1 (32)	42.5 (90)
Ethnicity			
Black	2.8 (6)	0.9 (2)	1.9 (4)
White	44.8 (95)	13.7 (29)	31.1 (66)
Mixed-race	52.4 (111)	12.3 (26)	40.1 (85)
Educational level			
Secondary education	10.8 (23	3.3 (7)	7.5 (16)
Technical education	39.2 (83)	7.1 (15)	32.1 (68)
Professional education	33.0 (70)	11.8 (25)	21.2 (45)
Specialization	11.8 (25)	2.8 (6)	9.0 (19)
Masters	5.2 (11)	1.9 (4)	3.3 (7)
Economic level			
1	0.5 (1)	0.0 (0)	0.5 (1)
2	22.2 (47)	6.6 (14)	15.6 (33)
3	61.3 (130)	15.6 (33)	45.8 (97)
4	12.7 (27)	4.2 (9)	8.5 (18)
5	2.8 (6)	0.5 (1)	2.4 (5)
6	0.5 (1)	0.0 (0)	0.5 (1)
Total	100.0 (212)	26.8 (57)	73.1 (155)

SD = standard deviation.

In the risk assessment for SARS-CoV-2 transmission, most of the positions or roles were classified as medium risk (51.9%, n = 110); however, in the individual assessment of risk of contagion, most of the workers classified their role or position as a high-risk activity (51.9%, n = 110). Considering the use of personal protective equipment, most of the participants wore surgical masks (63.7%, n = 135) throughout the working day. The accumulated training time spent on the prevention of SARS-CoV-2 infection was less than 120 minutes for most of the sample (67%, n = 142) (Table 2).

**Table 2:** Work conditions and risk perception evaluated in the sample of airport workers

Variables	Sex				p-value
	Female		Male		
	n	%	n	%	
Risk level, according to work position					
Low	20	9.4	57	26.9	0.97
Medium	30	14.2	80	37.7	
High	7	3.3	18	8.5	
Personal perception of risk					
Low	6	2.8	17	8.0	0.90
Medium	20	9.4	59	27.8	
High	31	14.6	79	37.3	
Use of face masks					
Never	2	0.9	0	0.0	0.2
Rarely	1	0.5	6	2.8	
Occasionally	6	2.8	30	14.2	
Most of the time	5	2.4	27	12.7	
Always	43	20.3	92	43.4	
Accumulated time training on the prevention of COVID-19					
No training	0	0.0	3	1.4	0.31
< 2 h	42	19.8	100	47.2	
> 2 h	15	7.1	52	24.5	
Total	57	26.9	155	73.1	

COVID-19 = coronavirus disease 2019.

### INCIDENCE AND CLINICAL PRESENTATION OF SARS-COV-2 INFECTIONS

The incidence of workers with a positive SARS-CoV-2 RT-PCR test was 7.92% (95%CI 4.19-11.64). Most of them were men (n = 10), but with no significant difference between sexes (p = 0.46). Workers with a positive RT-PCR test result were followed for 21 days. The vast majority were asymptomatic (81.25%, n = 13). Only one worker developed dyspnea, but did not require oxygen or other clinical management.

### SEROCONVERSION

RT-PCR results were negative in 56.3% (n = 9) of the workers at day 7. However, 18.3% (n = 3) of the nasopharyngeal samples remained positive until the 21st day of follow-up. Chemiluminescence immunoassay results were positive in 75% (n = 12) of the workers at day 21 (Table 3).

**Table 3:** Results of real time-polymerase chain reaction (RT-PCR) and antibody tests in workers followed for 21 days

Worker	Day 7		Day 14		Day 21	
	RT-PCR	Chemiluminescence	RT-PCR	Chemiluminescence	RT-PCR	Chemiluminescence
1	Pos	Neg	Neg	Neg	Neg	Pos
2	Pos	Neg	Neg	Neg	Neg	Neg
3	Pos	Neg	Pos	Neg	Neg	Pos
4	Pos	Neg	Neg	Neg	Neg	Neg
5	Pos	Neg	Neg	Pos	Neg	Pos
6	Pos	Neg	Neg	Pos	Neg	Neg
7	Pos	Neg	Pos	Pos	Neg	Pos
8	Pos	Neg	Neg	Pos	Neg	Neg
9	Pos	Pos	Neg	Pos	Neg	Pos
10	Pos	Neg	Neg	Neg	Neg	Pos
11	Pos	Neg	Pos	Pos	Neg	Pos
12	Pos	Pos	Neg	Neg	Neg	Pos
13	Pos	Neg	Pos	Pos	Pos	Pos
14	Pos	Neg	Pos	Pos	Pos	Pos
15	Pos	Neg	Pos	Pos	Pos	Neg
16	Pos	Neg	Pos	Pos	Pos	Pos

Neg = negative; Pos = positive; RT-PCR = reverse transcriptase-polymerase chain reaction.

### RISK FACTORS FOR SARS-COV-2 IN THE WORKPLACE

In the bivariate analysis, socioeconomic level was associated with positive RT-PCR test results (Fisher = 14.08; p = 0.03176). No associations were observed between the RT-PCR result and variables related to personal protective equipment, risk level, and risk perception (p > 0.05).

The Poisson regression model found that workers who had longer commutes had 1.02 times more risk of a positive RT-PCR result than those who spent less time commuting. There were no associations between mode of transport and RT-PCR results. The model also found that workers whose partners worked remotely had a protective factor. Workers with partners working from home had 4.5 times less risk of presenting a positive SARS-CoV-2 test. On the other hand, variables related to handwashing frequency and contact with passengers did not have any association with the measured outcomes (Table 4).

**Table 4:** Regression model for risk factors in the transmission of COVID-19 among airport workers

Variable	Adjusted RR	95%CI		p-value
		Inferior	Superior	
Age	0.984	0.920	1.052	0.635
Ethnicity	1.143	0.653	2.000	0.640
Sex	1.325	0.414	4.244	0.636
Educational level	1.162	0.628	2.150	0.632
Socioeconomic strata	1.712	0.746	3.929	0.205
# people in the household	0.965	0.566	1.647	0.897
# people in the household who work	0.973	0.379	2.498	0.954
People in the household working remotely	0.222	0.064	0.776	0.018
Duration of commute (home-workplace-home)	1.021	1.002	1.041	0.029
Cardiovascular diseases	0.939	0.186	4.748	0.939
Handwashing frequency	0.658	0.194	2.240	0.504
Mode of transport	1.342	0.796	2.262	0.269
Contact with passengers	2.160	0.274	16.881	0.465

95%CI = 95% confidence interval; COVID-19 = coronavirus disease 2019; RR = risk ratio.

## DISCUSSION

Globalization has increased passenger mobilization and merchandise trade, which are crucial for the functioning of the economy and for the development of science and technology. However, it also involves risks associated with the transmission of infectious diseases on aircrafts and airport terminals. This study detected an incidence of 7.92% in a population of airport workers. All workers who had a positive RT-PCR test result for SARS-CoV-2 displayed no symptoms, and none of their family and work contacts were infected. In addition, this study observed that workers who had longer commutes had 1.02 times more risk of having a positive RT-PCR result.

To our knowledge, this is the first work reporting on SARS-CoV-2 infection in airport workers. These findings are relevant to the extent that this is a population rarely studied in epidemic contexts. On the other hand, the finding that none of the participants developed clinical symptoms is striking. This is relevant because asymptomatic carriers of this virus represent a risk due to the lack of diagnosis and isolation, and these are potential transmitters of the virus in pre-shipment or through their contacts via multiple modes of transmission, including air, droplets, direct contact, and surfaces.^23^ Early identification and isolation of asymptomatic carriers can help prevent transmission to contacts such as work colleagues and passengers and also avoid possible outbreaks in the workplace.

In our analysis, most people who tested negative were young; this result is similar to the age distribution of confirmed cases reported by other studies.^24,25^ However, other studies have shown that age is not a protective factor.^26,27^

Regarding risk factors, our study observed that workers who spent more time commuting had a higher risk of a positive RT-PCR test result than those who had shorter commutes. Although our findings did not identify an association between mode of transport and commute duration, it is broadly known that citizens who use public transport tend to spend more time in traffic than those who use private modes of transport. Considering the pandemic, some authors have shown an association between the use of public transport and SARS-CoV-2 transmission.^28^ Moreover, different cities in China have identified the role of public transport in disseminating the infection in early February.^29,30^ However, to date, limited empirical data quantifies the risk of acquiring SARS-CoV-2 associated to suspended aerosols in public transport.^28^

In this study, we found a high frequency of mask-wearing (78.8%). This is striking because the WHO guideline for the widespread use of respiratory protection for the general community was not released until April 2020.^31^ Although it was not associated as a protective factor in our study, recent studies have shown that the use of a face mask reduces the risk of infection with SARS-CoV-2. A recently published meta-analysis found that the use of these types of attachments can result in a reduction in the risk of infection (odds ratio [OR] 0.15 [95%CI 0.07-0.34]).^32^

No associations were found with other working condition variables such as position, mode of transport, or duration of the working day. However, the behavior of risk level variables according to the work position and to individual perception was noticeable. Although no correlation was found between these variables, the individual perception of risk was higher than the evaluation for each position. These results are related to the time when this study was conducted; the level of knowledge on how the transmission of this virus happens in work environments outside the clinic was still poor.

Studies that evaluated risk perception related to work, regarding COVID-19, found that this level of self-perceived risk could be predicted by sex (women), area of residence, and whether or not they had children.^33^ On the other hand, this level of self-perceived risk has been reported to favor the adoption of behaviors and conducts that reduce the risk of infection.^34^

The results of this study had some limitations. First, the number of employees included in the study does not represent all airport employees, due to the study model being applied to a specific population at a defined time and place; simultaneous measurements of exposure and disease limited the possibility of making inferences about causality.^35^ Second, the OR associations may underestimate relationships, particularly considering that the frequency of presentation of SARS-CoV-2 infection in this population was low. Finally, the conditions in which samples were taken and the ability of RT-PCR to identify viral RNA in the first days of infection should be stated, since these may have occurred even though they were previously acknowledged and minimized by the training of the research team in the collection, packaging, and processing of samples. The generation of information that allows the implementation of strategies to protect workers, considering the pandemic, will have short- and long-term impacts; these workers and their occupational risk may be affected by the current or future pandemics. Additionally, more efficient control plans for pandemic management at the airport level cause a less severe economic impact on airports.^36^

In conclusion, the present study investigated the work factors related with COVID-19 infection in a high-risk population, since these workers have person-to-person contact with travelers that could be asymptomatic carriers of infectious diseases with a pandemic potential. Our result indicates the risk of asymptomatic infection by SARS-CoV-2 in airport workers. The control of risk factors could help prevent a new outbreak and ensure the protection of airport workers and their contacts.
